# Multiple endocrine neoplasia type 2

**DOI:** 10.1186/1750-1172-1-45

**Published:** 2006-11-14

**Authors:** Francesca Marini, Alberto Falchetti, Francesca Del Monte, Silvia Carbonell Sala, Isabella Tognarini, Ettore Luzi, Maria Luisa Brandi

**Affiliations:** 1 Regional Center for Hereditary Endocrine Tumors, Department of Internal Medicine, University of Florence, Florence, Italy; 2 DeGene Spin-off, Department of Internal Medicine, University of Florence, Florence, Italy

## Abstract

Multiple Endocrine Neoplasia Type 2 (MEN2) is a rare hereditary complex disorder characterized by the presence of medullary thyroid carcinoma (MTC), unilateral or bilateral pheochromocytoma (PHEO) and other hyperplasia and/or neoplasia of different endocrine tissues within a single patient. MEN2 has been reported in approximately 500 to 1000 families worldwide and the prevalence has been estimated at approximately 1:30,000. Two different forms, sporadic and familial, have been described for MEN2. Sporadic form is represented by a case with two of the principal MEN2-related endocrine tumors. The familial form, which is more frequent and with an autosomal pattern of inheritance, consists of a MEN2 case with at least one first degree relative showing one of the characteristic endocrine tumors. Familial medullary thyroid carcinoma (FMTC) is a subtype of MEN2 in which the affected individuals develop only medullary thyroid carcinoma, without other clinical manifestations of MEN2. Predisposition to MEN2 is caused by germline activating mutations of the *c-RET *proto-oncogene on chromosome 10q11.2. The *RET *gene encodes a single-pass transmembrane tyrosine kinase that is the receptor for glial-derived neurotrophic growth factors. The combination of clinical and genetic investigations, together with the improved understanding of the molecular and clinical genetics of the syndrome, helps the diagnosis and treatment of patients. Currently, DNA testing makes possible the early detection of asymptomatic gene carriers, allowing to identify and treat the neoplastic lesions at an earlier stage. In particular, the identification of a strong genotype-phenotype correlation in MEN2 syndrome may enable a more individualized treatment for the patients, improving their quality of life. At present, surgical treatment offers the only chance of cure and therefore, early clinical and genetic detection and prophylactic surgery in subjects at risk are the main therapeutic goal.

## Definition

Multiple Endocrine Neoplasia Type 2 (MEN2) (OMIM 171400) is a rare hereditary complex disorder characterized by the presence of medullary thyroid carcinoma (MTC), unilateral or bilateral pheochromocytoma (PHEO) and other hyperplasia and/or neoplasia of different endocrine tissues within a single patient. Two different forms, sporadic and familial, have been described for MEN2. Sporadic form is represented by a case with two of the principal MEN2-related endocrine tumors, while the familial form, which is more frequent and with an autosomal pattern of inheritance, consists of a MEN2 case with at least one first degree relative showing one of the endocrine characterizing tumors.

MEN2 includes three subtypes: MEN2A, MEN2B and Familial Medullary Thyroid Carcinoma (FMTC).

## Epidemiology

MEN2 has been reported in approximately 500 to 1000 families worldwide and the prevalence has been estimated at approximately 1:30,000. MEN2A accounts for more than 80% of all MEN2 cases.

## Clinical description, diagnostic methods, treatments

### MEN2A

This variant of the disease is characterized by the presence of medullary thyroid carcinoma (MTC), unilateral or bilateral pheochromocytoma (PHEO) (in more than 50% of cases) and primary hyperparathyroidism (PHPT) resulting from parathyroid cells hyperplasia or adenoma (15 to 30% of cases) [1]. Medullary thyroid carcinoma is generally the first manifestation of MEN2A. In MEN2A families, the biochemical manifestations of MTC appear between 5 and 25 years of age [2]. Rare variants of MEN2A can be associated with paraneoplastic syndromes such as cutaneous lichen amyloidosis or excessive production of corticotrophin. The lichenoid skin lesions are usually located over the upper portion of the back and may appear before the onset of MTC [3]. In addition, some patients with MEN2A develop Hirschsprung's disease (HD) that is characterized by the absence of autonomic ganglion cells within the distal colonic parasympathetic plexus, resulting in chronic obstruction and megacolon.

### MEN2B

This subtype is the most aggressive MEN2 variant. It accounts for about 5% of all cases of MEN2. MEN2B is characterized by the earlier occurrence (usually ten years earlier than in MEN2A) of a more aggressive MTC, PHEO (40–50% of cases) and multiple neuromas, and/or diffuse ganglioneuromatosis of the gastroenteric mucosa (about 40% of cases), but not hyperparathyroidism. Ganglioneuromatosis of the gastrointestinal tract is responsible for abdominal distension, megacolon, constipation or diarrhea. Patients with MEN2B manifest developmental abnormalities, such as decreased upper/lower body ratio, skeletal deformations (kyphoscoliosis or lordosis), joint laxity, marfanoid habitus and myelinated corneal nerves.

Morbidity and mortality is higher in patients with MEN2B than in patients with MEN2A.

### Familial Medullary Thyroid Carcinoma (FMTC)

In FMTC, MTC is the only clinical feature. This form, according to the statement of the International RET Mutation Consortium [4], refers to the occurrence of MTC only in at least four affected members within the same family. In FMTC, the clinical course of MTC is more benign than that in MEN2A and MEN2B, and the prognosis is relatively good in most cases [5].

### Medullary Thyroid Carcinoma (MTC)

This tumor, originating from the parafollicular calcitonin-producing cells (C-cells), is the first clinical manifestation in most MEN2 kindreds and occurs in all MEN2 patients. MTC usually occurs in a decreasing order of severity in MEN2B, MEN2A and FMTC, respectively [6]. MTC originates as multifocal C-cell hyperplasia, whose progression to MTC is extremely variable and may take several years [7]. MTC exhibits a natural tendency to local metastases (central and lateral, cervical and mediastinal lymph nodes) and, often, to distant lesions (liver, bones and lungs) [8].

In MEN2A patients, the biochemical manifestations of MTC generally appear between the age of 5 and 25 years. If these individuals are untreated, MTC can manifest as a neck mass or neck pain at age of 15 to 20 years. Diarrhea may occur in patients with spread metastases from MTC, in association with high plasma calcitonin concentrations.

In MEN2B patients, the MTC is more aggressive and usually develops about a decade earlier. Individuals with MEN2B who do not undergo thyroidectomy at age of one year, are likely to develop metastatic MTC at early age.

MTC generally correlates with increased circulating levels of calcitonin (basal, or stimulated by pentagastrin and calcium, or both), thus calcitonin can be considered as a specific tumor marker for MTC (normal basal value <10 pg/ml). In the 1970s, the introduction of specific tests to evaluate calcitonin secretion under stimulation with pentagastrin or calcium enhanced the possibility for earlier detection of MTC [9]. Similarly, an elevated calcitonin serum level after surgery can be a sign of persistent, recurrent or generalized MTC [5,10].

Surgery is the treatment of choice for MTC, in both MEN2A and MEN2B patients. It consists of total thyroidectomy and lymph node dissection of at least the central compartment, that should ideally be performed before the age of possible malignant progression. Fine-needle biopsy and evaluation of serum calcitonin level (basal, and two and five minutes after stimulation with calcium) have been shown to help the preoperative diagnosis of MTC. Calcitonin stimulation test can be useful for presurgical assessment of MTC stage, as there is a positive correlation between basal and stimulated calcitonin levels, and Tumor-Nodes-Metastasis (TNM) classification. Moreover, imaging procedures, such as ultrasonography, computed tomography (CT) or magnetic resonance imaging (MRI) may be used to determine tumor's extension and possible distant metastases [11].

### Pheochromocytoma (PHEO)

This catecholamine-producing tumor of the adrenal gland appears in about 50% of the MEN2A and MEN2B patients. Pheochromocytomas in MEN2 are almost always benign but tend to be bilateral in 50–80% of cases. Generally, PHEO is the first clinical manifestation of the disease in 25% of cases (after MTC in 40%); in 35% of cases, MTC and PHEO are diagnosed at the same time [5,12-14]. PHEO may account for hypertension, episodic headache, palpitations, nervousness, sweating and blanching of the skin due to excessive synthesis of epinephrine, norepinephrine and dopamine by the chromaffin cells of the adrenal gland. Prior to surgery, the presence of a functioning PHEO should be excluded by appropriate biochemical analysis in all MEN2A and MEN2B individuals. If PHEO is detected, adrenalectomy should be performed before thyroidectomy or any other surgical intervention, in order to avoid intraoperative catecholamine crisis. Adrenal gland function can be easily assessed by 24 hours measurements of urinary excretion of catecholamines and their metabolites (norepinephrine, epinephrine, metanephrine and vanillymandelic acid (VMA)); this screening is recommended on an annual basis. Once the biochemical diagnosis of PHEO has been made, CT and MRI can be used to localize the tumor, with a specificity of about 70%. ^123^I-meta-iodobenylguanidine scintigraphy (iodine-123-MIGB) of the whole body is helpful in patients with suspected multifocal or extra-adrenal PHEO, with a sensitivity of about 80% and a specificity of nearly 100%. The treatment of PHEO is surgical laparoscopy excision. Life-long follow-up after surgery is strongly recommended. Long-term drug treatment with α and β adrenergic blockers should be considered only in patients with unresectable (for various reasons) tumor.

### Primary Hyperparathyroidism (PHPT)

PHPT occurs in 20 to 30% of MEN2A patients and is asymptomatic in most cases (6). The diagnosis is made by biochemical screening showing an elevated serum parathyroid hormone (PTH) and calcium concentrations. Treatment of PHPT in MEN2 is surgical, by subtotal parathyroidectomy or total parathyroidectomy with autotransplantation of normal fresh or cryopreserved tissue in the sternocleidoid muscle or in the forearm [5]. All individuals who have undergone subtotal parathyroidectomy or total parathyroidectomy with autotransplantation need to be monitored for possible recurrences.

## Molecular genetics of MEN2

### *c-RET *oncogene and its protein

In 1993, MEN2 syndrome was strongly associated with activating mutations of the *c-RET *proto-oncogene [15,16]. This gene is located on the pericentromeric region of chromosome 10 (10q12.2). It has twenty-one exons and encodes a membrane tyrosine kinase receptor protein named RET. The RET protein is a subunit of a multimolecular complex that binds growth factors of the glial derived neurotrophic factor (GDNF) family. It consists of i) an extracellular portion composed by six different domains (four cadherine-like domains, a calcium-binding site and a cysteine-rich domain), ii) a single-pass transmembrane domain and iii) an intracellular portion containing two distinct tyrosine kinase domains. The intracellular domain of RET contains at least 12 autophosphorylation sites. RET phosphorylated tyrosines serve as docking sites for intracellular signaling proteins.

Approximately 98% of MEN2 patients bear germline mutations of *c-RET *[6]. MEN2 mutations are localized in exons 10, 11, 13, 14, 15 and 16. Very recently, mutations in exon 8 have been found in a FMTC family [17]. Mutations of *c-RET *in the cysteine-rich domain and in the tyrosine kinase domains result all in a constitutive tyrosine kinase activation of the mutant receptor.

### Genotype-phenotype correlation

MEN2 syndrome exhibits a strong genotype-phenotype correlation. Missense mutations at one of six cysteines in the extracellular cysteine-rich domain of RET (609, 611, 618, 620, 630 at exon 10 and 634 at exon 11) are responsible for the majority of cases of MEN2A (93–98%) and a majority of FMTC (80–96%). Eighty-five percent of MEN2A patients have a codon 634 mutation, particularly C634R, strictly associated with the occurrence of PHEO and/or primary hyperparathyroidism [18]. Only 30% of FMTC patients have a codon 634 mutations; in general, the mutations in FMTC patients are distributed among the six cysteine codons. FMTC and rare cases of MEN2A have been also associated with missense mutations in the intracellular domain of RET, such as codons 768, 790 and 791 at exon 13, codons 804 and 844 at exon 14 and codon 891 at exon 15 [19].

Rare mutations in codon 631 at exon 10 have also been found in FMTC. In contrast, most of MEN2B cases are associated with mutations in the intracellular tyrosine kinase receptor domains of RET: more than 95% of MEN2B patients bear the M918T mutation at exon 16, while about 5% of MEN2B patients harbor the A883F substitution at exon 15 [20,21].

Recently, other infrequent missense mutations at exons 14, 15 and 16 (respectively at codons 804 and 806, 904, and 922) have been found in MEN2B patients [11].

The oncogenic mechanisms of different *RET *mutations seem to be dependent on the site of the amino acid change. Cysteine substitution with several other residues, in the cysteine-rich domain, is believed to prevent the formation of intramolecular disulfide bonds, enabling ligand-independent receptor dimerization and resulting in a constitutive kinase activation. Thus, cysteine point mutations of MEN2A and FMTC have a "gain of function" effect on RET. When triggered by its ligand, wild type RET receptors are induced to dimerize and this dimerization activates RET kinase activity and signal transduction [22]. In MEN2A and FMTC, mutated RET receptors, as a consequence of substitution of one extracellular cysteine residue, are constitutively dimerized independently of ligand. Mutations at cysteine 634 have shown a stronger transforming ability than mutations at other extracellular cysteines. In contrast, the great majority of mutations in MEN2B cases affect one of two intracellular tyrosine kinase domains. However, little is known at present about the mechanisms of RET activation by mutations in the tyrosine kinase domains. RET phosphorylated tyrosines interact with the docking protein FSR2 causing the downstream activation of the mitogen-activated protein kinase (MAPK) signaling cascade [23]. Thus, mutations at this level may determinate an alteration of the regulation of MAPK pathways [24,25]. Recently, a microarray expression analysis of PHEO and MTC tissues from patients with MEN2A and MEN2B demonstrated different gene expression profiles, possibly explaining the more aggressive nature of MEN2B [26].

## Genetic screening and management

MTC represents the first clinical manifestation of MEN2 syndrome and is the principal cause of morbidity and mortality. Early identification of MTC, mostly in MEN2B patients, is important because of its tendency to metastasize in early age. In 1970s, the development of specific tests to evaluate calcium or pentagastrin-stimulating release of calcitonin allowed the detection of MTC in its earliest stage [9]. Nevertheless, it was the discovery of the correlation between mutations of *c-RET *and MEN2 syndrome in 1993 that has opened a new era in the early recognition and clinical management of the affected and at-risk individuals. The DNA-based testing of the *c-RET *gene can be easily performed on a blood sample at any age. It offers the opportunity for early identification of the *c-RET *germline mutations, thus contributing to the reduction of morbidity and mortality of MEN2 syndrome. In fact, the early recognition of the mutant gene carriers makes possible the prevention and cure of MTC, by performing a prophylactic thyroidectomy before the clinical expression of the tumor. This test is also of importance to detect and thus, to reduce the risk of an unsuspected PHEO. Moreover, the aggressiveness of MTC correlates with the specific *c-RET *codon mutation and this strong genotype-phenotype correlation ulteriorly contributes to the clinical management of patients. Specific *c-RET *mutations, in fact, are associated to peculiar clinical phenotypes and thus to different course and prognosis of the disease. During the Seventh International Multiple Endocrine Neoplasia Meeting in Gubbio in 1999 [6], the risk of MTC has been stratified in three categories according to the mutations of *c-RET*:

1) Children with MEN2B and/or *c-RET *codon 883, 918, 922 mutations have the highest risk of aggressive MTC (level 3) and should undergo a total thyroidectomy with central node dissection, within the first six months.

2) Children with any *c-RET *codon 611, 618, 620 or 634 mutations have a high risk of MTC (level 2); in this case, a total thyroidectomy should be performed before age of five years, with or without central node dissection.

3) Children with *c-RET *codon 609, 768, 790, 791, 804 and 891 mutations have a less aggressive and slowly growing MTC (level 1) and may be operated at a later stage. Some clinicians recommend a prophylactic thyroidectomy by the age of five, while others suggest thyroidectomy by age of ten. A periodic pentagastrin-stimulated test with thyroidectomy, at the first abnormal test result, has also been proposed.

Nevertheless, for all three groups, a more aggressive neck dissection should be performed if there is an evidence of lateral lymph nodes involvement [27]. For individuals bearing other *c-RET *known mutations, no specific recommendations can be made at present, as there is no sufficient experience with these kindreds. More recently, Cohen and Moley have suggested a prophylactic thyroidectomy with autotransplantation of parathyroids as a primary preventive measure for all individuals with identified germline *c-RET *mutation [28].

Genetic information can also be useful to assess the risk of developing PHEO. Individuals with *c-RET *codon 609, 611, 618, 620, 630, 634, 790, V804L, 883, 918 or 922 mutations should be routinely screened for PHEO by annual determinations of fractionated urinary and free plasma metanephrines and catecholamines. In contrast, it is reasonably acceptable that development of PHEO is unlikely in patients with codon 768 and V804M mutations [29].

## Genetic counseling

MEN2 is a monogenic disorder and, according to its autosomal dominant pattern of inheritance, each affected individual has a 50% probability of transmitting the gene defect to progeny, independently by sex.

## Prognosis

MEN2 is a rare hereditary cancer disease expressing a variety of aggressive endocrine and non-endocrine tumors. Although uncommon, this syndrome is important to be early recognized because the gene mutations confer a high risk of multiple aggressive primary tumors occurring at very young ages. Medullary thyroid carcinoma occurs in all patients with MEN2 and it can be lethal. In 1993, the discovery of MEN2 responsible gene strengthened the possibility of early identification of the affected and at-risk individuals. In the last decades, the increasing knowledge on the molecular and clinical features of MEN2 syndrome, together with the availability of genetic tests, greatly increased the opportunities of intervention and, consequently, lead to reduced morbidity and mortality. At the moment, surgical treatment offers the only chance of cure and therefore early clinical and genetic detection and prophylactic surgery are the goal in subjects at risk.

## Perspectives

Further studies on the molecular pathways of *c-RET *gene and its protein will help to design novel and more individualized therapeutic modalities based on genetic information [30]. In fact, although the knowledge about mechanisms of tumor development in patients with MEN2 has grown tremendously, much work lies ahead. The final goal is to offer patients with *c-RET *germline mutations an optimal cancer prevention and treatment program.
